# Primary Podocytopathies After COVID-19 Vaccination

**DOI:** 10.1016/j.ekir.2021.12.023

**Published:** 2021-12-24

**Authors:** Sjoerd A.M.E.G. Timmermans, Matthias H. Busch, Myrurgia A. Abdul-Hamid, Leon A.M. Frenken, Albert-Jan Aarnoudse, Pieter van Paassen

**Affiliations:** 1 Department of Nephrology and Clinical Immunology, Maastricht University Medical Center, Maastricht, The Netherlands; 2 Department of Pathology, Maastricht University Medical Center, Maastricht, The Netherlands; 3 Department of Internal Medicine, Zuyderland Medical Center, Heerlen, The Netherlands; 4 Department of Internal Medicine, Catharina Hospital Eindhoven, Eindhoven, The Netherlands

**Keywords:** COVID-19, FSGS, kidney biopsy, minimal change disease, podocytopathies, vaccinations

Ever since the global administration of various severe acute respiratory syndrome coronavirus 2 (SARS-CoV-2) vaccines in response to coronavirus disease 2019 (COVID-19), temporal associations between glomerulopathies and vaccination have been reported.^1^ Minimal change disease (MCD)^1^, ^2^, ^3^ and perhaps tip lesion variant of focal segmental glomerular sclerosis^2^ along the spectrum of primary podocytopathies may develop shortly after vaccination against COVID-19 (i.e., <6 weeks), suggesting a direct role of the vaccine. Likewise, MCD has been associated with immunization against other viruses. Reports describe the disease course of individual patients and, thus, do not prove causation.

By October 31, 2021, a total of 24,062,166 vaccines have been administered in The Netherlands, with approximately 85% of Dutch citizens aged 18 years and above being fully vaccinated against COVID-19.^4^ BNT162b2 (Pfizer/BioNTech), with 18,465,082 doses (76.8%) administered, and ChAdOx1 nCoV-19 (also known as AZD1222, AstraZeneca), with 2,780,131 doses (11.6%) administered, are the most widely used vaccines in The Netherlands.^4^ The Dutch vaccination program provides an opportunity to investigate “COVID-19 vaccine-associated podocytopathy” in the southeast part of The Netherlands using data from the Limburg Renal Registry,^5^ a regional observational cohort of patients who underwent a native kidney biopsy. This multicenter study sought to define the incidence of *de novo* podocytopathies in 2021, that is, MCD and tip lesion variant of focal segmental glomerular sclerosis, as compared with a historical cohort, including 5 consecutive years before the COVID-19 pandemic (i.e., from January 2015 to December 2019). Moreover, clinical and morphologic characteristics, COVID-19 vaccination status, and clinical outcomes were evaluated. The methods are detailed in the Supplementary Methods. Here, we provide data on clustering of primary podocytopathies in time, pointing to a potential association with COVID-19 vaccination.

In 2021, a total of 140 patients who underwent a native kidney biopsy until October 31 at the latest were recruited; 12 patients with a repeated kidney biopsy were excluded. Of 128 enrolled patients, 14 (10.9%) presented with a *de novo* primary podocytopathy (Table 1); 11 and 3 patients presented with MCD and tip lesion variant of focal segmental glomerular sclerosis, respectively. The incidence of *de novo* primary podocytopathies was higher as compared with 5 consecutive years before the COVID-19 pandemic (14 [10.9%] of 128 vs. 23 [4.2%] of 548 “first” native kidney biopsies; Fisher exact test, *P* < 0.01). The higher incidence of MCD, in particular, explained this increase (11 [8.6%] of 128 vs. 19 [3.5%] of 548, respectively; Fisher exact test, *P* = 0.02). The annual incidence of MCD did not differ in the period before the COVID-19 pandemic (Supplementary Results), similar to previous observations.^5^

**Table 1:** Baseline characteristics of patients diagnosed with primary podocytopathies in 2021

No.	Age, yr	Sex	Pattern	Podocyte-associated IgG	Vaccine (before presentation)	Onset, wk	Dose	Baseline parameters			
								SCr, mg/dl	SAlb, g/l	UP, g/d	MCCS, 0–10
COVID-19 vaccine-associated podocytopathy											
M10321	64	F	MCD	−	ChAdOx1 nCoV-19	1	1	0.8	11.8	6.9	1
M12621	34	M	MCD	−	BNT162b2	4	2	1.0	16.2	10.4	0
M13721	74	M	MCD	+	BNT162b2	6	2	1.8	16.0	7.4	0
M13921	47	M	FSGS	−	BNT162b2	<2	2	0.8	20.4	4.5	0
Podocytopathy unrelated to COVID-19 vaccination											
M00621	36	M	MCD	−	−			4.9	15.5	41.6	0
M00821	46	F	MCD	+	−			1.0	10.5	5.6	0
M01021	19	M	MCD	−	−			0.7	13.8	3.5	1
M01621	22	F	MCD	+	−			0.6	10.6	6.8	0
M02821	59	F	MCD	−	−			0.7	15.0	4.3	2
M05321	22	M	MCD	−	−			1.2	3.3	7.8	0
M05621	54	M	FSGS	−	−			1.0	22.5	3.1	0
M06121	75	M	FSGS	−	BNT162b2	>6	N/A	1.0	21.1	6.9	1
M13621	67	F	MCD	−	BNT162b2	>6	N/A	1.4	27.1	8.8	3
M14021	28	F	MCD	+	BNT162b2	>6	N/A	0.7	15.8	8.7	0

F, female; FSGS, tip lesion variant of focal segmental glomerular sclerosis; M, male; MCCS, Mayo Clinic Chronicity Score; MCD, minimal change disease; N/A, not available; SAlb, serum albumin; SCr, serum creatinine; UP, proteinuria.

Conversion factor for unit SCr in mg/dl to μmol/l, ×88.4.

The higher incidence of *de novo* primary podocytopathies in 2021 seemed to be associated with COVID-19 vaccination. A total of 3 of 11 patients (27%) with MCD and 1 of 3 patients (33%) with tip lesion variant of focal segmental glomerular sclerosis were considered to have COVID-19 vaccine-associated podocytopathy (Table 1); secondary etiologies, such as, drugs and malignancies, were ruled out. None of these patients had a medical history of “symptomatic” SARS-CoV-2 infection. Patients invariably presented with nephrotic syndrome, with median serum albumin level of 16.1 (range, 11.8–20.4) g/l. The onset of symptoms varied from 1 week after the first dose of ChAdOx1 nCoV-19 to <6 weeks after the second dose of BNT162b2, corroborating previous reports.^1^, ^2^, ^3^ The patient who received ChAdOx1 nCoV-19 noticed recurrent edema after the second dose; at that point, prednisolone was postponed because of concerns regarding vaccine efficacy. It is therefore possible that some of the patients who received BNT162b2 may have had proteinuria between the first and second doses but had not been evaluated in that interim. Minimal chronic changes, that is, Mayo Clinic Chronicity score 0 to 1, were found on kidney biopsy results, indicating a short duration of disease.

The mechanism of COVID-19 vaccine-associated podocytopathy remains to be established. Most cases have been associated with the mRNA-based BNT162b2,^6^ although other vaccines, including the adenoviral-vectored ChAdOx1 nCoV-19, have been implicated as well. BNT162b2 and ChAdOx1 nCoV-19 stimulate robust T cell responses.^7^ Deregulated T cell activation has been linked to primary podocytopathies^8^ and may occur on the background of COVID-19 vaccination. ChAdOx1 nCoV-19 induces a stronger T cell response than BNT162b2 after the first dose.^7^ MCD, indeed, has been reported to occur within days after the first dose of ChAdOx1 nCoV-19,^1^ whereas the onset of disease varies in patients who received BNT162b2.^1^, ^2^, ^3^ Remarkably, 1 patient had podocyte-associated punctate polyclonal IgG deposits but no electron dense deposits on kidney biopsy (M13721; Figure 1), pointing to B cell activation and production of autoantibodies that target the slit diaphragm.^9^ Thus, both cellular and humoral immune responses are conceivable for primary podocytopathy to occur after COVID-19 vaccination.

**Figure 1:**
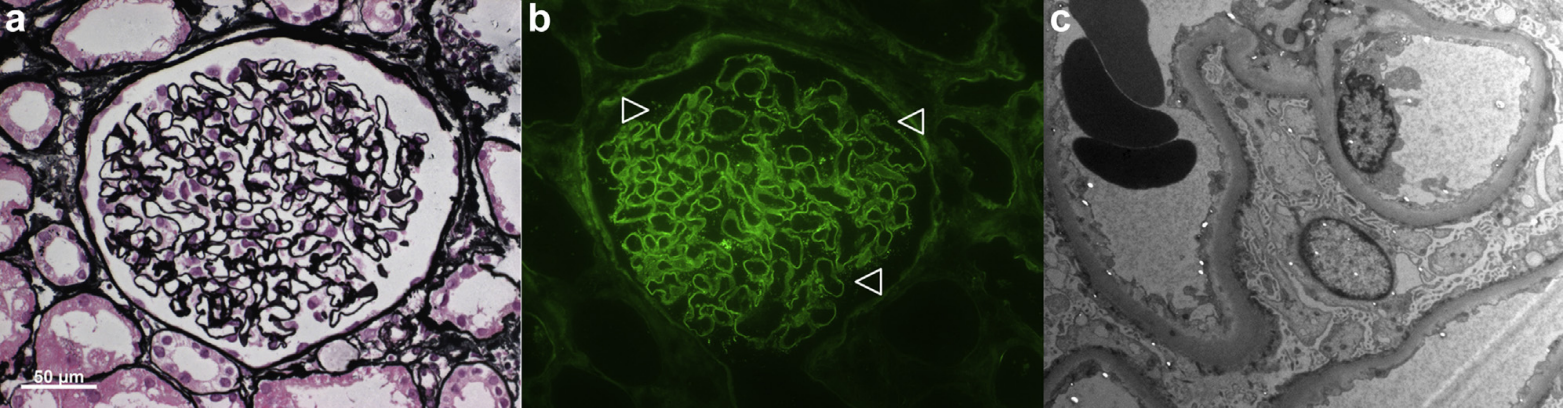
Minimal change disease with podocyte-associated punctate polyclonal IgG deposits on kidney biopsy. (a) Normal appearing glomerulus on light microscopy (Jones methenamine silver; original magnification ×400). (b) Podocyte-associated punctate polyclonal IgG deposits on immunofluorescence microscopy (IgG; original magnification ×400), the staining of which is minimal as compared to that seen in membranous nephropathy. (c) No electron dense deposits were found on electron microscopy (original magnification ×4200).

Furthermore, 2 patients with COVID-19 vaccine-associated podocytopathy were treated with high-dose prednisolone and 2 patients who denied prednisolone were treated conservatively. Identical to previous reports,^1^^,^^2^ 1 patient on prednisolone achieved a complete remission (M10321 followed for 4 months), resembling the disease course of MCD unrelated to COVID-19 vaccination.^8^ It is too early to know the response of the other patient on prednisolone (M13721 followed for 1 month). None of the patients on best supportive care, including renin-angiotensin system blockade, remitted at last follow-up (M12621 followed for 2 months; M13921 followed for 1 month). At present,^1^^,^^2^ although further studies are required to determine clinical outcomes, the outcome of patients with COVID-19 vaccine-associated podocytopathy treated with prednisolone seems favorable.

Taken together, this case series provide data on primary podocytopathies after COVID-19 vaccination. This year’s higher incidence of *de novo* primary podocytopathies and, in particular, MCD, in the Limburg Renal Registry and the temporal association between onset of disease and COVID-19 vaccination suggest causality. The overwhelming benefits of vaccination should be recognized, and therefore, nephrologists should be aware of this association as global vaccination efforts continue and third-dose vaccinations are being deployed.

## Disclosure

All the authors declared no competing interests.

## Supplementary Material

Supplementary File (PDF)

**Table S1.** Patients enrolled in the Limburg Renal Registry.

**Supplementary Methods.**

**Supplementary Results.**

**Supplementary References.**
