# Supplementary material for: Primary Podocytopathies After COVID-19 Vaccination
Source: Kidney Int Rep. 2021 Dec 24;7(4):892–4. doi: 10.1016/j.ekir.2021.12.023 (PMC8702635; doi:10.1016/j.ekir.2021.12.023)
Supplement: Supplementary File (PDF) [file mmc1.pdf]

Supplementary Material, including Methods and Results.

Sjoerd A.M.E.G. Timmermans,[1] M.D., Matthias H. Busch,[1] M.D., Myrurgia A. Abdul-Hamid,[2] M.D., Leon A.M. Frenken,[3] M.D., Ph.D., Albert-Jan Aarnoudse,[4] M.D., Ph.D., Pieter van Paassen,[1] M.D., Ph.D.; for the Limburg Renal Registry.

[1]Dept. Nephrology and Clinical Immunology and [2]Dept. Pathology, Maastricht University Medical Center, Maastricht, The Netherlands. [3]Dept. Internal Medicine, Zuyderland Medical Center, Heerlen, The Netherlands. [4]Dept. Internal Medicine, Catharina Hospital Eindhoven, Eindhoven, The Netherlands.

Supplementary Methods.

The Limburg Renal Registry is a regional observation cohort, including patients who have had a native kidney biopsy, in the south-east of the Netherlands.[1] Patients enrolled between January 1, 2021, and October 31, 2021, were recruited; patients with a repeated kidney biopsy were excluded. In addition, a historical cohort, covering 5 consecutive years prior to the Covid-19 pandemic (from January 2015 until December 2019), was included. The incidence of de novo primary podocytopathies, that is, minimal change disease (MCD) and tip lesion variant of focal segmental glomerulosclerosis (FSGS), was studied in both cohorts. MCD was defined as normal appearing glomeruli on light microscopy with extensive foot process effacement and no electron dense deposits on electron microscopy. Tip lesion variant of FSGS was defined as endocapillary foam cells at the tubular poli with adhesion or confluence of podocytes with parietal and/or tubular epithelium at the tubular outlet and extensive foot process effacement; collapsing and perihilar variants of FSGS,[2] considered different entities as compared to MCD and tip lesion variant of FSGS,[3] were excluded. The Mayo Clinic Chronicity Score was deployed to grade chronic changes on kidney biopsy.[4]

Covid-19 vaccine-associated podocytopathy was considered in patients with a temporal association between vaccination and disease onset (i.e., <6 weeks).[5-8]

***Supplementary Results*.**

Table S1. Patients enrolled in the Limburg Renal Registry. The incidence of de novo primary podocytopathies as a whole (Fisher's exact test, $P<0.01$) and MCD (Fisher's exact test, $P=0.02$) but not tip lesion variant of FSGS was higher in 2021 as compared to the period prior to the Covid-19 pandemic; the annual incidence of de novo primary podocytopathies as a whole, MCD, and tip lesion variant of FSGS did not differ in the latter period.

		**Historical cohort**				
**Kidney biopsy**	**2021**	**2019**	**2018**	**2017**	**2016**	**2015**
Total	140	140	127	144	132	112
First biopsy	128	113	103	124	107	101
MCD	11	2	2	5	4	6
FSGS	3	0	2	0	1	1
Repeated biopsy	12	27	24	20	25	11

References

1. van Paassen P, van Breda Vriesman PJ, van Rie H*, et al.* Signs and symptoms of thin basement membrane nephropathy: a prospective regional study on primary glomerular disease-The Limburg Renal Registry. *Kidney Int* 2004; **66:** 909-913.

2. D'Agati VD, Fogo AB, Bruijn JA*, et al.* Pathologic classification of focal segmental glomerulosclerosis: a working proposal. *Am J Kidney Dis* 2004; **43:** 368-382.

3. D'Agati VD, Alster JM, Jennette JC*, et al.* Association of histologic variants in FSGS clinical trial with presenting features and outcomes. *Clin J Am Soc Nephrol* 2013; **8:** 399-406.

4. Sethi S, D'Agati VD, Nast CC*, et al.* A proposal for standardized grading of chronic changes in native kidney biopsy specimens. *Kidney Int* 2017; **91:** 787-789.

5. Li NL, Coates PT, Rovin BH. COVID-19 vaccination followed by activation of glomerular diseases: does association equal causation? *Kidney Int* 2021; **100:** 959-965.

6. Klomjit N, Alexander MP, Fervenza FC*, et al.* COVID-19 vaccination and glomerulonephritis. *Kidney Int Rep* 2021.

7. Salem F, Rein JL, Yu SM*, et al.* Report of Three Cases of Minimal Change Disease Following the Second Dose of mRNA SARS-CoV-2 COVID-19 Vaccine. *Kidney Int Rep* 2021; **6:** 2523-2524.

8. Leclerc S, Royal V, Lamarche C*, et al.* Minimal Change Disease With Severe Acute Kidney Injury Following the Oxford-AstraZeneca COVID-19 Vaccine: A Case Report. *Am J Kidney Dis* 2021; **78:** 607-610.
